# Testing the effects of gentle vibrotactile stimulation on symptom relief in fibromyalgia

**DOI:** 10.1186/s13075-019-1932-9

**Published:** 2019-06-14

**Authors:** Jesus Pujol, Daniel Ramos-López, Laura Blanco-Hinojo, Guillem Pujol, Héctor Ortiz, Gerard Martínez-Vilavella, Josep Blanch, Jordi Monfort, Joan Deus

**Affiliations:** 1 MRI Research Unit, Department of Radiology, Hospital del Mar, Passeig Marítim 25-29, 08003 Barcelona, Spain; 2 Centro Investigación Biomédica en Red de Salud Mental, CIBERSAM G21, Barcelona, Spain; 3 Department of Project and Construction Engineering, Universitat Politècnica de Catalunya (UPC), Barcelona, Spain; 4 Rheumatology Department, Hospital del Mar, Barcelona, Spain; 5 Department of Clinical and Health Psychology, Autonomous University of Barcelona, Barcelona, Spain

**Keywords:** Fibromyalgia, Somatosensory system, Pain, Vibrotactile stimulation, Sensory balance

## Abstract

**Background:**

Sensory disturbances in fibromyalgia extend beyond nociception. It has been proposed that imbalance in the mutual competition between painful input and non-painful sensory activity may, to a significant extent, account for the augmented subjective perception of pain. In this context, non-nociceptive somatosensory stimulation could arguably attenuate fibromyalgia symptoms by restoring the sensory balance. We specifically tested the effect of vibrotactile stimulation on symptom relief in fibromyalgia patients with a randomized, double-blind, sham-controlled, crossover clinical trial.

**Methods:**

Seventy-seven female patients were randomized and data from 63 valid cases were analyzed. Active intervention involved extensive body stimulation with gentle mechanical vibrations administered during 3 h at night for 3 weeks, and the placebo effect was controlled using identical instruments to simulate an alternative treatment option. The primary outcome measure combined pain, fatigue, and complaints of poor cognition.

**Results:**

Vibrotactile stimulation was significantly superior to sham in alleviating fibromyalgia symptoms globally. However, univariate analyses showed that the effect was not universal. Benefits were perceived on unpleasant somatic sensations such as generalized pain and fatigue, but not on poor cognition, anxiety, and depression. Vibrotactile stimulation was notably well tolerated and sleep quality significantly improved despite the fact that vibrations were administered at night.

**Conclusions:**

Results thus provide new evidence that non-nociceptive somatosensory stimulation may favorably act upon altered somatosensory balance in fibromyalgia. From a clinical perspective, both the degree of improvement and the easy application of our proposal would seem to support a potential role for vibrotactile stimulation in the symptomatic treatment of fibromyalgia.

**Trial registration:**

ClinicalTrials.gov registration number NCT03227952. Registered 24 July, 2017.

## Background

Fibromyalgia has been a controversial disorder in some medical contexts due to the subjective nature of its symptoms. Patients indeed complain of generalized pain, fatigue, unrefreshing sleep, and poor cognition in the absence of an observable organic cause [1]. Nevertheless, current methods for studying neural function offer new opportunities to explore subtle clinical phenomena. One such tool that contributes to characterizing the pathophysiology of fibromyalgia is functional MRI [2–4]. By means of functional connectivity and task activation approaches, we have recently observed functional alterations beyond the nociceptive domain which would suggest a weak integration of other sensory inputs contributing to clinical pain in fibromyalgia [5, 6]. Other research has provided data consistent with such a downregulation of the non-nociceptive component of somatosensory processing [7–12].

It has been proposed that imbalance in the mutual competition between painful inputs and non-painful sensory activity may, to a significant extent, account for the augmented subjective perception of pain and body discomfort [6]. In this context, non-nociceptive somatosensory stimulation could arguably attenuate fibromyalgia symptoms by restoring the sensory balance, regardless of whether non-painful sensory activity alteration is a primary phenomenon or a by-product of originally facilitated (or deficiently filtered) nociceptive signals. There are empirical studies demonstrating the beneficial effects of treatments based on physical/sensory stimulation that do not directly aim at the nociceptive system. Tested procedures showing a range of success include, for instance, physical exercise and movement-based therapies, hydrotherapy, and peripheral nerve stimulation [11, 13–17]. Interestingly, the benefits of non-nociceptive stimulation contrast with the paradoxically poor efficacy of genuine analgesic drugs in relieving fibromyalgia pain [13, 15, 18].

One way to selectively stimulate the non-nociceptive component of the somatosensory system is by using mechanical vibrations. Vibrotactile stimuli are captured by a variety of widespread skin and musculoskeletal tissue receptors and transmitted via large-diameter myelinated fibers separately from the nociceptive pathway [19]. Experimental studies interestingly suggest that fibromyalgia pain can be effectively modulated by vibrotactile stimuli [20]. Moreover, there are empirical studies specifically testing the potential usefulness of vertical oscillating platforms that have reported optimistic results [21–25]. However, there have been no previous studies controlling for the possible placebo effects of vibration-based treatments applied to fibromyalgia patients.

In our study, we tested the effect of vibrotactile stimulation on symptom relief in fibromyalgia patients with a randomized, double-blind, sham-controlled, crossover clinical trial. The intervention involved extensive body stimulation with gentle mechanical vibrations administered during 3 h at night for 3 weeks, and the placebo effect was controlled using identical instruments to simulate an alternative treatment option. The primary outcome was change in the key symptoms of fibromyalgia combining pain, fatigue, and complaints of poor cognition.

## Materials and methods

This study was conducted in accordance with the principles expressed in the Declaration of Helsinki and was approved by the Ethical Committee of Clinical Research of the Parc de Salut Mar of Barcelona (reference no. 2016/6932/I). All patients provided written informed consent. The trial was designed according to CONSORT recommendations [26] and was registered at the US National Institutes of Health (ClinicalTrials.gov), with identifier number NCT03227952 and title “Study of the effectiveness of vibrotactile sensory stimulation in fibromyalgia patients”.

### Participants

Patients were recruited from the Fibromyalgia Unit of Barcelona’s Hospital del Mar - Parc de Salut Mar between September 2017 and May 2018. A total of 166 patients clinically diagnosed with fibromyalgia were contacted through a consecutive order based on clinical visit schedules (JB and JM). One hundred and seventeen patients agreed to be screened for eligibility and were fully briefed on the study and the corresponding inclusion/exclusion criteria. Seventy-seven patients were eventually randomized following the exclusion of 23 patients who did not meet study criteria and 17 patients who declined to participate.

#### Eligibility criteria

Inclusion criteria were based on the following factors: female, aged 18 to 65 years, diagnosed by a specialist in fibromyalgia in accordance with the American College of Rheumatology classification and diagnostic criteria [1, 27], the patient did not suffer from any other disorder that might account for the pain, chronic use treatments in stable doses, a full understanding of the study, and an express commitment to compliance. Criteria to exclude patients were generalized inflammatory articular or rheumatic disease; severe or non-stable medical, endocrine, or neurological disorder; psychotic disorder or drug abuse; evidence of poor compliance; and events that could relevantly interfere with the trial.

Intervention effects were evaluated when added to ongoing treatments. Changes in the previously prescribed treatments and procedural therapies (e.g., nerve blocks or joint injections) were forbidden throughout the trial. Only variations in the daily dose of the habitual analgesics were allowed.

### Study design

This randomized, double-blind, sham-controlled, two-period crossover clinical trial measured the effect of vibrotactile sensory stimulation versus sham treatment on symptom relief in fibromyalgia patients. All patients underwent a period of vibrotactile stimulation and a period of sham with complete counterbalancing, half of them in that sequence and the other half first receiving sham treatment. Both treatments lasted 3 weeks and were separated by a 2-week washout period.

### Study interventions

**Fig. 1:**
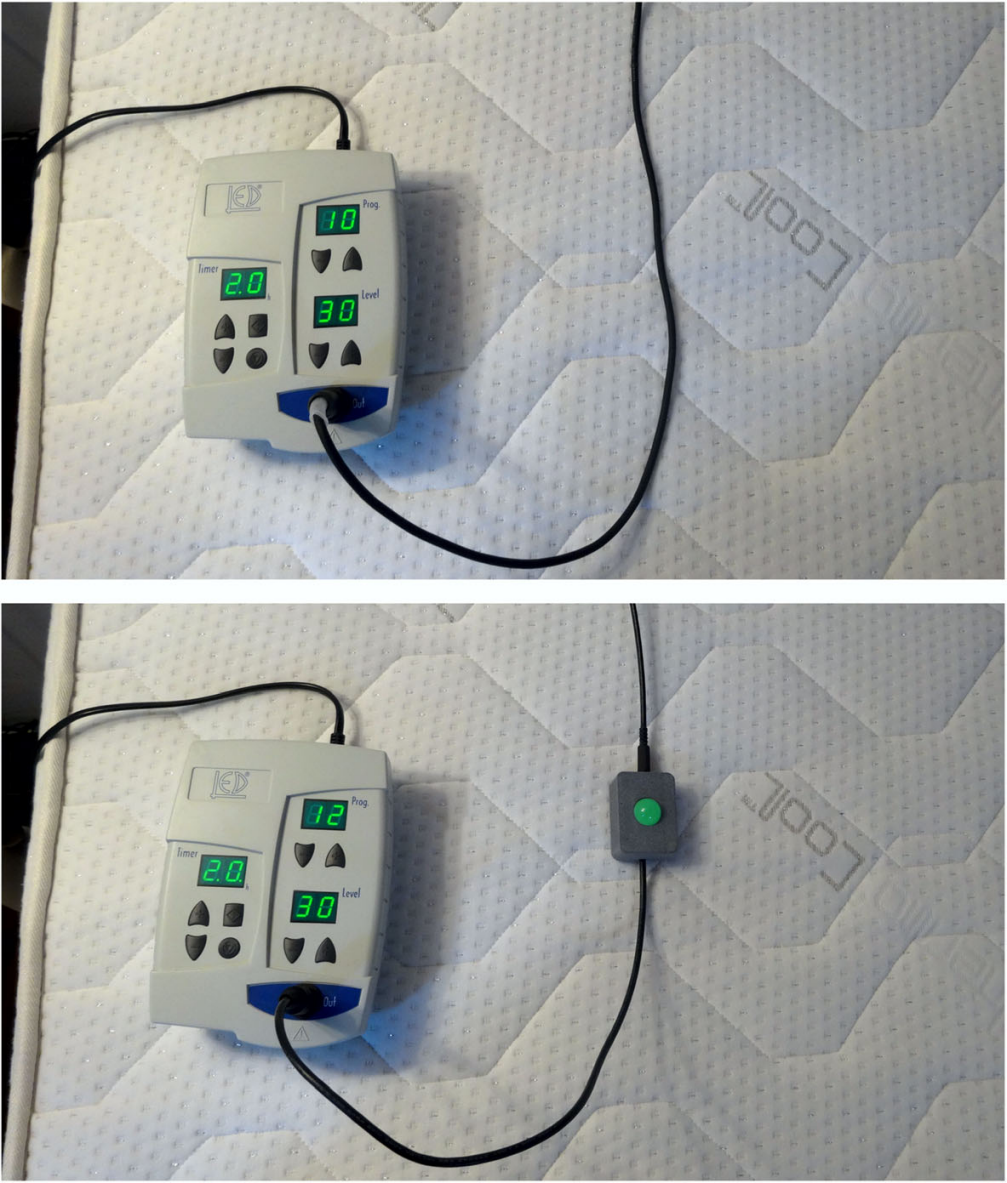
Instruments used to administer vibrotactile stimulation (above) and sham (bottom). An electronic engine controlled stimuli delivery. Mechanical vibrotactile stimuli were generated using 6 vibration motors fitted into a standard mattress. Sham treatment was applied using identical instruments, with an electrical signal turning on a pilot light to indicate that the (simulated) treatment was operative

The active treatment involved whole-body sensory stimulation with mechanical stimuli of vibrotactile (pallesthetic) type at a relatively high rate, low intensity, and long duration administered at sleep time. To generate mechanical vibrotactile stimuli, 6 vibration motors (i.e., conventional electric motors with an eccentric mass fixed to the rotor) were fitted into a standard mattress (80 by 190 cm) symmetrically positioned to fully cover it, with the exception of the area in which the patient’s head lay while sleeping. The 6 vibration motors generated a slowly varying spectrum of frequencies ranging from 2 to 90 Hz, which was mechanically transmitted to the whole mattress. An electronic programmer was built to control stimulus delivery for duration and intensity (Fig. 1). The whole vibration system was manufactured by LED SpA (Aprilia, Italy).

The treatment involved the application of vibrotactile stimulation with a total daily duration of 3 h distributed within a period of 2 h at bedtime and 1 h prior to getting up. The intensity was set to 30% of power (see below) at bedtime and 45% prior to getting up. Patients were instructed to turn on the system at bedtime and try to get to sleep naturally. The system was programmed to automatically stop 2 h later. The following morning, once the patients had naturally awoken, they were to set the device to run for a further hour and remain in their beds. Patients were allowed to fall asleep during this period.

Intensity and frequencies of vibrations delivered were measured in real conditions using a tri-axial accelerometer specifically designed to measure the exposure of workers to vibrations transmitted to the whole body (CESVA AC033, CESVA Instruments, Barcelona) according to the International Organization for Standardization’s norm ISO 2631-1. The accelerometer was calibrated using the CESVA CV211 multi-frequency vibration calibrator. Vibration spectrum was determined using VSHOOTER VBS1T vibration analysis camera (Synergys Technologies, Altkirch, France).

The measurements obtained indicated that the normalized vibration intensity applied to the body at 30% of power in a 3-h exposure was 0.03 m/s^2^, and for 3 h at 45%, it was 0.04 m/s^2^. Such energy delivery is much lower than the daily occupational exposure permitted for whole-body vibrations at work (1.15 m/s^2^) according to current European and Spanish regulations (Spanish law RD 1311/2005). And also below the limit from which adopting procedures for risk prevention at work is recommended (0.5 m/s^2^). When the stimulus was set at 30% of power, dominant frequencies were widely distributed with a peak at 22 Hz and secondary peaks from 4 to 90 Hz. When the stimulus was set at 45% of power, the dominant frequency peaked at 30.6 Hz instead.

Sham treatment was applied using identical instruments and with power and duration programmed identically. However, in this case, the output was not the signal activating the vibration motors, but rather an electrical signal turning on an incorporated pilot light indicating that the (simulated) treatment was operating (Fig. 1). The patient was told that “the study involved the comparison of 2 treatment options; vibration versus magnetic waves, both of which are potentially effective treatments in fibromyalgia.” However, patients were clearly informed that study interventions might or might not be effective in the context of a clinical trial. The participants were also informed that the interventions had no known relevant health risks under the applied conditions.

Special attention was paid to ensuring blinding. One blind researcher collected all outcome measures. Also, the assessment interviews were entirely structured and the interaction between the data collector and patients was strictly limited to the questions of interest. No other conversations were permitted during the interview. Prior to the study, patients formally agreed not to interact with the researcher collecting outcome measures. Another researcher was available throughout the study to be consulted by patients in the event of doubt, adverse effects, or any kind of incident. Assessment and monitoring were carried out in different centers with no interaction during the evolution of the study.

#### Randomization

Patients were randomly assigned to the intervention sequence (vibrotactile stimulation first or sham first) with an equal allocation ratio (1:1). The randomization scheme was generated by means of the Web site Randomization.com http://www.randomization.com. The system generated a unique number for each randomized patient. The randomization schedule was stratified in blocks of 8. A single independent researcher was aware of the randomization process and randomization information (LBH). This researcher was in charge of telephoning each patient on the day to start the interventions to communicate the device (real or sham) to be connected in each phase.

### Outcome measures

The primary outcome was “change in fibromyalgia’s key symptoms rated by 101-point numerical rating scales (NRS).” The effect on pain, fatigue, and “cognitive symptoms” was jointly analyzed using a repeated measures multivariate analysis of variance (MANOVA) including separate NRS scores. In addition, participants rated intervention effects providing a single global NRS score for all three symptoms (pain, fatigue, and cognitive symptoms) considered together. This rating was given by participants as a direct estimation of percentage (%) improvement of their symptoms after the interventions.

The rationale for using such a combination of symptoms is their relevance in characterizing fibromyalgia as a syndrome, in addition to “waking unrefreshed” [1]. “Waking unrefreshed” was not considered within the primary outcome as the study’s intervention was applied during the night and we had no a priori knowledge of its effects on sleeping comfort.

Pain, fatigue, and cognitive symptoms were individually rated at study baseline as the overall symptom severity during the last month. Upon their first visit, patients were specifically instructed and trained to rate symptoms using NRS, in which 0 was no symptoms and 100 the worst possible. Due to the large dispersion of spontaneous pain and subjective symptom measures in fibromyalgia patients [18, 28, 29], treatment effects on both vibrotactile stimulation and sham were evaluated at the end of the study using the initial scores as a reference. To assist symptom rating, patients were instructed to firstly evaluate the effects of the intervention as a percentage change (improvement or worsening). Percentage ratings were then transformed into absolute NRS values. Patients evaluated each treatment effect globally as sustained (> 1 week) improvement (or worsening) attained during each 3-week treatment period.

Secondary outcomes included the following: (i) “Unrefreshed Sleep,” patients rated the quality of sleep by indicating the severity (i.e., how much of a problem) of awakening tired or unrefreshed using 101-point NRS. (ii) “Daily Activity Impact,” the interference of fibromyalgia in their daily life activities and quality was similarly quantified using 101-point numerical ratings. (iii) “Emotional Distress,” assessed on the Hospital Anxiety and Depression Scale (HADS) [30, 31] with separate rating for anxiety and depression. (iv) The Fibromyalgia Impact Questionnaire (FIQ) [32], total score. Secondary outcome measures were obtained both before and after each treatment period.

### Sample size

We estimated that a total of 58 valid patients would be required to detect a difference between allocation groups with a two-tailed *α* of 0.05, a “1-β” of 0.80, symptom reduction standard deviation of 20 points (on 101-point ratings), and symptom reduction difference of 15 points (relevant symptom reduction differences have been reported as ranging from 10 to 20 in previous studies [33, 34]). If a potential case loss of 20% is assumed, our aim was to randomize a minimum of 73 patients (77 cases were finally randomized).

### Statistical analysis

The change regarding the corresponding baseline (pre minus post) was used as a measure of treatment response for pain, fatigue, and cognitive symptoms. Differences between sensory stimulation and sham effects were jointly tested for all three key symptoms using repeated measures MANOVA. Within-subject effects were then estimated with the repeated measures model for each individual (pain, fatigue, and cognitive symptoms) rating. To test intervention order effects, a between-subject factor with two levels (sensory stimulation first or sham first) was added to the repeated measures model. Univariate analyses to compare intervention effects within and between allocation groups were carried out using paired *t*-test and two-sample *t*-test, respectively.

Secondary outcomes were analyzed similarly. Participants receiving at least 1 day of either study intervention were included in adverse effect analyses. Proportions of participants with adverse effects were analyzed using Fisher’s exact test. All reported *p* values are two-tailed. All analyses were conducted using SPSS software, version 20.

## Results

**Fig. 2:**
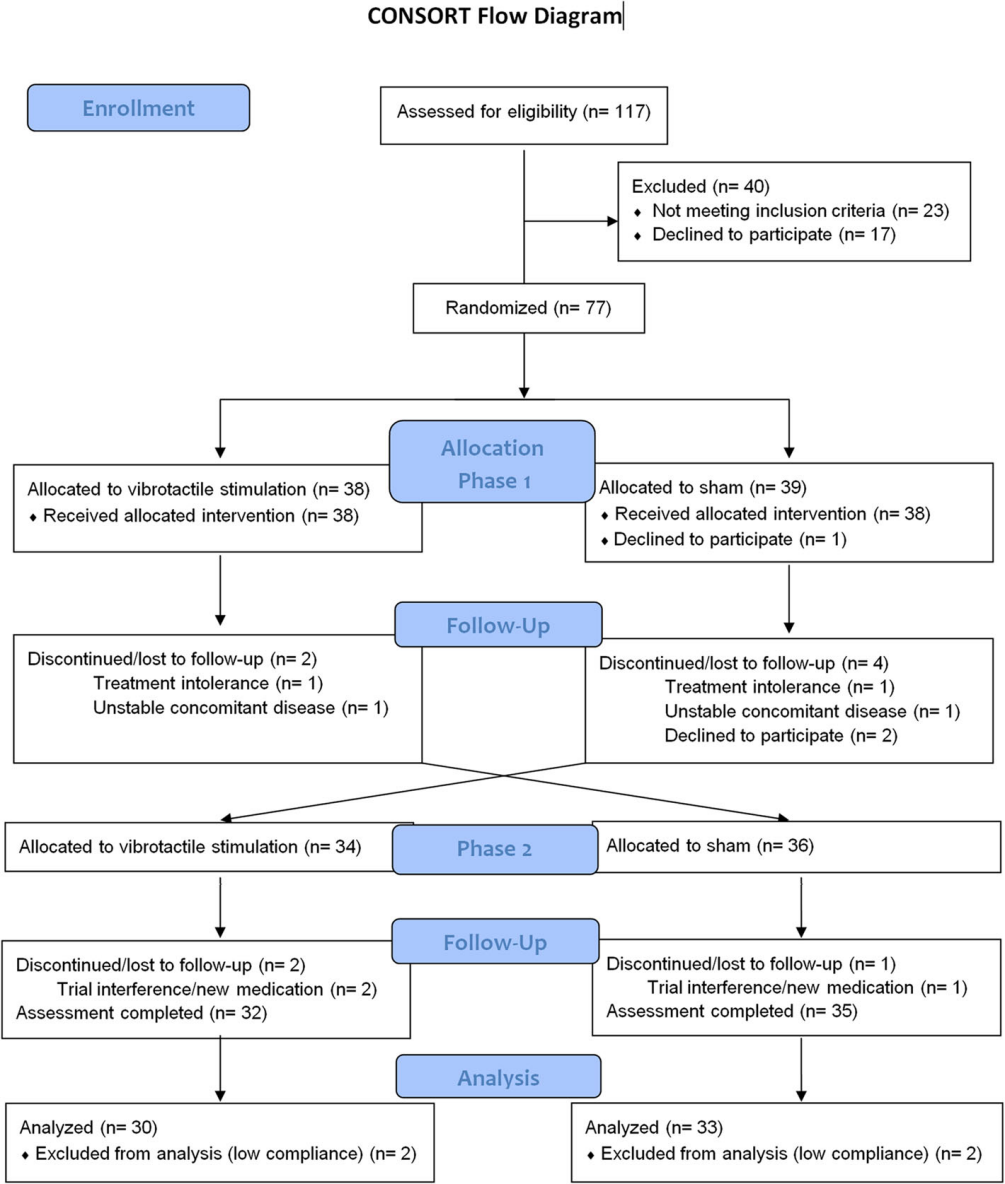
CONSORT flow diagram

Of the 77 patients randomized, 10 patients discontinued the study and 4 were excluded due to low treatment compliance. The final analyzed sample included 63 valid patients, of whom 30 belonged to the “sham first” group and 33 to the “vibrotactile stimulation first” group (Fig. 2). Baseline demographic and clinical characteristics are detailed in Table 1.

**Table 1:** Clinical characteristics (*n* = 63)

Age, years, mean (SD)	53.7 (8.1)
Sex, females, *n* (%)	63 (100)
Height, cm, mean (SD)	161 (6)
Weight, kg, mean (SD)	69.2 (13.6)
Hand-dominance, *n* (%) right-handers	56 (89)
Education level, years, mean (SD)	14.1 (3.9)
Illness duration, mean (SD), years since diagnosis	7.7 (5.8)
Fibromyalgia Impact Questionnaire*, mean (SD)	63.6 (15.1)
General perception of health**, mean (SD)	33.1 (17.6)
Hospital Anxiety and Depression Scale (HADS)	
Anxiety, mean (SD)	11.2 (4.1)
Depression, mean (SD)	9.8 (4.4)
Stable medication regime	
NSAIDs, *n* (%)	29 (46)
Opioids (tramadol), *n* (%)	24 (38)
Paracetamol, *n* (%)	34 (54)
Gabapentin, *n* (%)	25 (40)
Antidepressants	
SSRI, *n* (%)	18 (29)
SNRI, *n* (%)	20 (32)
Others, *n* (%)	14 (22)
Anxiolytics/hypnotics (benzodiazepines), *n* (%)	42 (67)

*Fibromyalgia Impact Questionnaire (FIQ), maximum score, 100. **According to the 36-Item Short-Form Health Survey, maximum score, 100. Several patients were taking more than one medication. *NSAIDs* non-steroidal anti-inflammatory drugs, *SSRI* selective serotonin reuptake inhibitor, *SNRI* serotonin-norepinephrine reuptake inhibitor

### Primary outcome

Repeated measures MANOVA including the variable change (pre minus post) in NRS ratings for pain, fatigue, and cognitive symptoms showed a significant within-subject overall effect (vibrotactile stimulation versus sham) with *F* = 4.0 and *p* = 0.012.

Significant differences between vibrotactile stimulation and sham were also obtained for the percentage improvement rated for pain, fatigue, and cognitive symptoms globally. Changes in the global symptom score for sham showed mean ± SD of 25.7% ± 30.7% and vibrotactile stimulation 45.2% ± 33.9%, *t* = 3.4 and *p* = 0.001.

### Fibromyalgia key symptoms

**Fig. 3:**
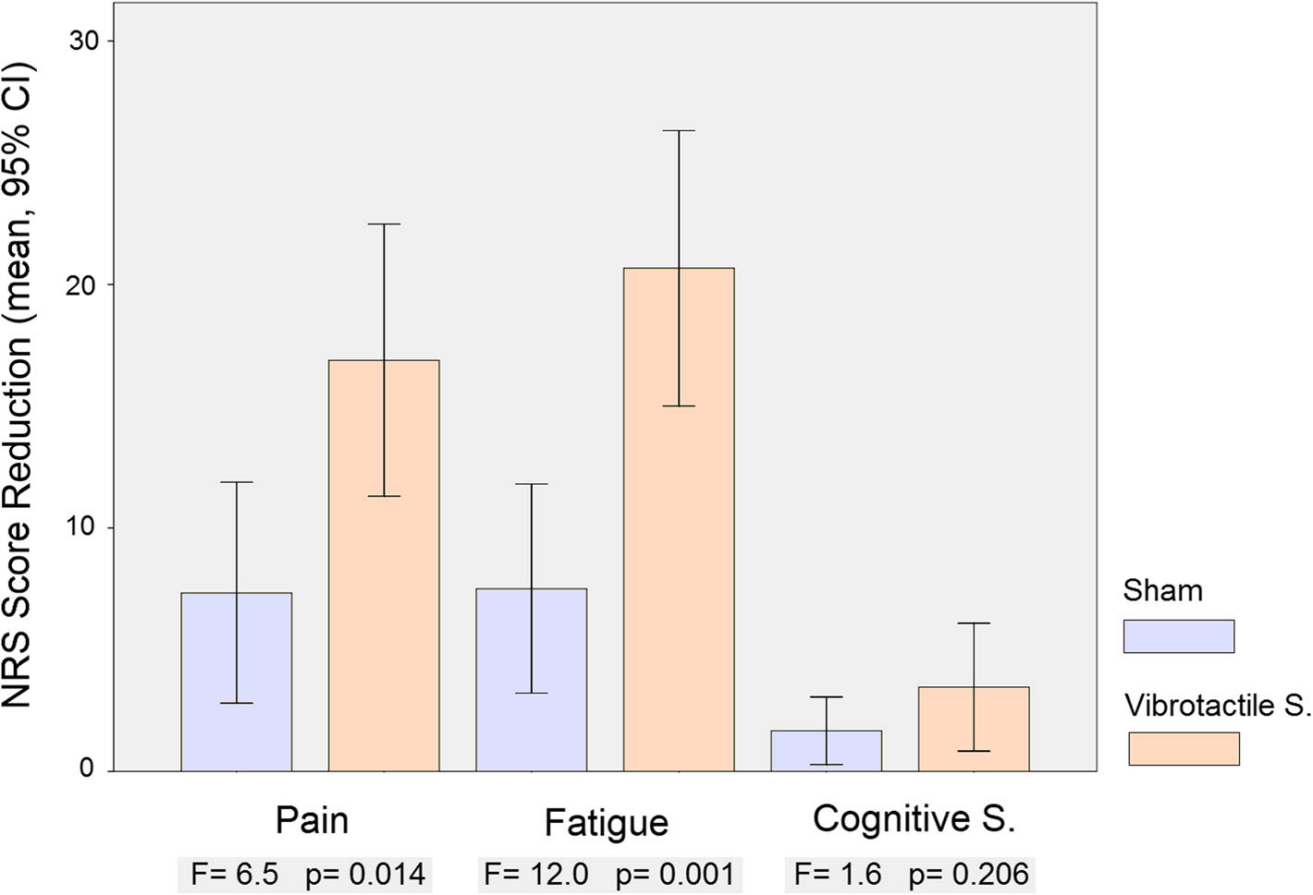
Bar graphs showing NRS score reduction for the three fibromyalgia key symptoms

Univariate contrasts for the three fibromyalgia key symptoms showed score change indicating a symptom improvement significantly higher for vibrotactile stimulation than for sham in pain and fatigue, but not in cognitive symptoms (Table 2, Fig. 3).

**Table 2:** Fibromyalgia key symptoms

All patients (*n* = 63)	Baseline (B_vs_)	Vibrotac. S. (VS)	Baseline (B_sham_)	Sham	(B_vs_ − VS) > (B_sham_ − Sham)
Pain	71.3 ± 13.1	54.4 ± 23.5	69.9 ± 15.1	62.5 ± 21.0	*F* = 6.5 *p* = 0.014
Fatigue	73.8 ± 13.7	53.1 ± 26.4	71.8 ± 15.5	64.3 ± 22.9	*F* = 12.0 *p* = 0.001
Cognitive symptoms	65.5 ± 18.1	62.0 ± 21.0	65.8 ± 18.2	64.1 ± 19.4	*F* = 1.6 *p* = 0.206
Vibrotactile S first (*n* = 33)	Baseline (B_1_)	Vibrotac. S. (VS)	Baseline (B_2_)	Sham	(B_1_ − VS) > (B_2_ − Sham)
Pain	71.2 ± 10.4	48.0 ± 26.1	66.4 ± 16.3	62.8 ± 20.0	*t* = 4.0 *p* = 0.0003
Fatigue	74.4 ± 12.5	46.8 ± 27.8	69.1 ± 16.3	65.7 ± 21.9	*t* = 5.1 *p* = 0.00001
Cognitive symptoms	66.5 ± 18.0	61.9 ± 22.4	66.4 ± 18.1	63.9 ± 20.3	*t* = 0.9 *p* = 0.359
Sham first (*n* = 30)	Baseline (B_1_)	Sham	Baseline (B_2_)	Vibrotac. S. (VS)	(B_2_ − VS) > (B_1_ − Sham)
Pain	73.7 ± 12.9	62.2 ± 22.4	71.3 ± 15.7	61.3 ± 18.3	*t* = − 0.3 *p* = 0.783
Fatigue	74.7 ± 14.3	62.7 ± 24.2	73.1 ± 15.0	60.1 ± 23.4	*t* = 0.2 *p* = 0.842
Cognitive symptoms	65.2 ± 18.6	64.4 ± 18.8	64.4 ± 18.5	62.1 ± 19.7	*t* = 0.9 *p* = 0.382
	(B_1_ − VS) > (B_1_ − Sham)		(B_2_ − VS) > (B_2_ − Sham)		
Pain	*t* = 2.2 *p* = 0.034		*t* = 1.5 *p* = 0.149		
Fatigue	*t* = 2.9 *p* = 0.005		*t* = 2.3 *p* = 0.025		
Cognitive symptoms	*t* = 1.9 *p* = 0.071		*t* = − 0.1 *p* = 0.911

Table 2 shows within and between comparisons for allocation groups. A significant intervention order effect was identified. That is, the difference between vibrotactile stimulation and sham for pain and fatigue was significantly greater in the “vibrotactile stimulation first” group than in the “sham first” group (interaction “intervention by order” showing *F* = 8.8 and *p* = 0.004 for pain, and *F* = 10.7 and *p* = 0.002 for fatigue).

**Fig. 4:**
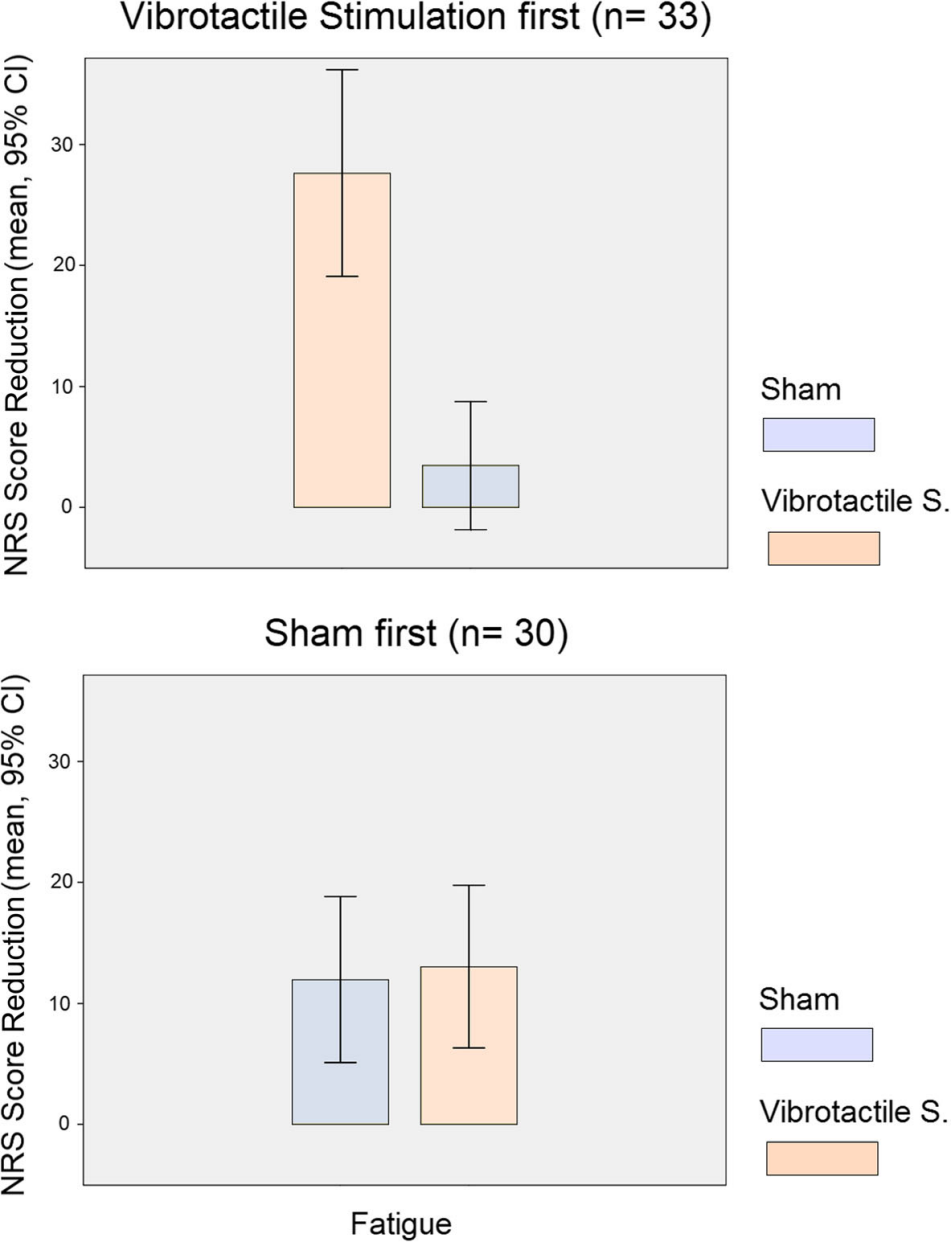
Bar graphs showing NRS score reduction for fatigue separately for each allocation group to illustrate the intervention order effect

Figure 4 illustrates the intervention order effect for fatigue ratings. There was a virtual absence of placebo effect in the sham condition when administered in the second period (*n* = 33; mean ± SD pre minus post NRS, 3.5 ± 14.9; *t* = 1.3 and *p* = 0.190). Interestingly, the effect of sham when administered first was similar to the effect of vibrotactile stimulation when administered secondly (*n* = 30; NRS 12.0 ± 18.4 versus 13.0 ± 18.0, *t* = − 0.2 and *p* = 0.842). By contrast, the differences between both interventions were notably large when vibrotactile stimulation was administered first (*n* = 33; NRS 27.6 ± 24.1 versus 3.5 ± 14.9, *t* = 5.1 and *p* = 0.00001).

Another carryover phenomenon was that the effect of vibrotactile stimulation, when administered first, did not completely disappear at week 5 (i.e., 2 weeks after the end of vibrotactile stimulation) (Table 2). For instance, NRS scores of fatigue at baseline showed a mean ± SD of 74.4 ± 12.5 and at week 5 a mean ± SD of 69.1 ± 16.3 (*n* = 33; difference = 5.2; *t* = 3.0 and *p* = 0.005). By contrast, differences in fatigue NRS ratings between baseline and week 5 were not significant in the “sham first” group (*n* = 30; baseline fatigue NRS scores 74.7 ± 14.3 versus week 5 scores 73.1 ± 15.0; difference = 1.6; *t* = 1.8 and *p* = 0.087). The interaction, however, was not significant (*F* = 3.4 and *p* = 0.068).

### Secondary outcomes

The advantage of vibrotactile stimulation over sham was also significant for restoring sleep quality. That is, the changes observed for the “Unrefreshed Sleep” variable generally paralleled the results of pain and fatigue (Table 3). As for disorder impact, the effect of vibrotactile stimulation had a non-significant tendency to be higher (compared with sham) for both measures (Daily Activity Impact and Fibromyalgia Impact Questionnaire). Vibrotactile stimulation had no significant effect on emotional distress assessed on the Hospital Anxiety and Depression Scale.

**Table 3:** Secondary outcome measures

All patients (*n* = 63)	Baseline (B_vs_)	Vibrotac. S. (VS)	Baseline (B_sham_)	Sham	(B_vs_ − VS) > (B_sham_ − Sham)
Unrefreshed Sleep	69.6 ± 17.8	55.1 ± 22.5	67.1 ± 18.5	60.3 ± 23.4	*t* = 2.2 *p* = 0.033
Daily Activity Impact	70.6 ± 17.9	67.9 ± 16.9	69.7 ± 17.8	69.8 ± 18.6	*t* = 1.7 *p* = 0.098
Anxiety (HADS)	10.9 ± 4.4	10.1 ± 3.9	11.0 ± 3.9	10.9 ± 4.3	*t* = 1.4 *p* = 0.164
Depression (HADS)	9.3 ± 4.7	9.3 ± 4.7	9.3 ± 4.4	9.3 ± 4.6	*t* = − 0.6 *p* = 0.955
FIQ	61.9 ± 16.2	54.8 ± 17.3	60.7 ± 17.9	58.1 ± 18.0	*t* = 1.9 *p* = 0.069
Vibrotactile S. first (*n* = 33)	Baseline (B_1_)	Vibrotac. S. (VS)	Baseline (B_2_)	Sham	(B_1_ − VS) > (B_2_ − Sham)
Unrefreshed Sleep	70.8 ± 16.8	52.7 ± 23.8	61.9 ± 20.6	61.9 ± 24.2	*t* = 4.4 *p* = 0.0001
Daily Activity Impact	68.9 ± 19.4	64.8 ± 18.6	66.6 ± 19.0	67.7 ± 20.3	*t* = 2.4 *p* = 0.020
Anxiety (HADS)	10.7 ± 4.7	10.0 ± 3.8	10.5 ± 4.2	10.7 ± 4.4	*t* = 1.1 *p* = 0.261
Depression (HADS)	9.8 ± 4.8	9.4 ± 4.5	9.0 ± 4.8	9.6 ± 4.7	*t* = 1.5 *p* = 0.148
FIQ	63.1 ± 14.0	54.1 ± 18.2	57.5 ± 19.0	58.3 ± 18.7	*t* = 3.4 *p* = 0.002
Sham first (*n* = 30)	Baseline (B_1_)	Sham	Baseline (B_2_)	Vibrotac. S. (VS)	(B_2_ − VS) > (B_1_ − Sham)
Unrefreshed Sleep	72.8 ± 14.1	58.6 ± 22.7	68.4 ± 19.1	57.6 ± 21.0	*t* = − 0.6 *p* = 0.527
Daily Activity Impact	73.2 ± 15.9	71.1 ± 16.7	72.4 ± 16.2	71.3 ± 14.3	*t* = − 0.7 *p* = 0.502
Anxiety (HADS)	11.6 ± 3.4	11.2 ± 4.2	11.2 ± 4.2	10.2 ± 4.0	*t* = 0.8 *p* = 0.427
Depression (HADS)	9.8 ± 3.9	8.9 ± 4.6	8.8 ± 4.6	9.1 ± 4.9	*t* = − 1.4 *p* = 0.171
FIQ	64.2 ± 16.3	57.8 ± 17.5	60.7 ± 18.4	55.6 ± 16.5	*t* = − 0.4 *p* = 0.753
	(B_1_ − VS) > (B_1_ − Sham)		(B_2_ − VS) > (B_2_ − Sham)		
Unrefreshed Sleep	*t* = 1.0 *p* = 0.336		*t* = 2.2 *p* = 0.029		
Daily Activity Impact	*t* = 1.4 *p* = 0.162		*t* = 1.3 *p* = 0.198		
Anxiety (HADS)	*t* = 0.3 *p* = 0.759		*t* = 1.8 *p* = 0.082		
Depression (HADS)	*t* = −0.6 *p* = 0.549		*t* = 0.5 *p* = 0.595		
FIQ	*t* = 0.7 *p* = 0.462		*t* = 1.6 *p* = 0.111		

*FIQ* Fibromyalgia Impact Questionnaire, *HADS* Hospital Anxiety and Depression Scale

### Additional “intent-to-treat” analysis

Although the aim of our study was to reflect actual treatment differences, an additional analysis was carried out including all patients with available outcome measures (*n* = 67). The results were almost identical for all variables with the exception of Daily Activity Impact and Fibromyalgia Impact Questionnaire. Differences between vibrotactile stimulation and sham for Daily Activity Impact rating in this analysis showed *t* = 2.1 and *p* = 0.041, and Fibromyalgia Impact Questionnaire score *t* = 2.0 and *p* = 0.049 (with data missing from one patient in the Fibromyalgia Impact Questionnaire), both in the direction of symptom improvement being higher for vibrotactile stimulation. It is noteworthy that, in the primary analysis, both variables showed only a tendency to statistical significance (Table 3).

### Adverse effects

Both vibrotactile stimulation and sham were generally well tolerated. 101-point NRS for discomfort showed a mean ± SD of 11.9 ± 23.6 in patients receiving vibrotactile stimulation and 6.1 ± 21.9 when receiving sham (*n* = 67; *t* = 1.7 and *p* = 0.099). Two additional patients were initially excluded due to excessive discomfort; one who failed to tolerate vibrotactile stimulation and the other failing to tolerate sham (Fig. 2). Six patients required the intensity of the stimulus to be reduced to 66% of the prescribed power due to discomfort; in 4 cases while being treated with vibrotactile stimulation and in 2 cases with sham (*χ*^2^ = 0.5 and *p* = 0.674). Therefore, night-time vibrotactile stimulation did not generally affect sleep in patients, but it did significantly improve sleep quality in the whole study group (Table 3).

## Discussion

Vibrotactile stimulation was significantly superior to sham in alleviating fibromyalgia symptoms globally. However, univariate analyses showed that the effect was not universal. Benefits were perceived on unpleasant somatic sensations such as generalized pain and fatigue, but not on poor cognition, anxiety, and depression. Vibrotactile stimulation was notably well tolerated and sleep quality significantly improved despite the vibrations being administered at night. The extent of symptom improvement may be sufficiently relevant to suggest a potential role for vibrotactile stimulation as symptomatic treatment in fibromyalgia. Moreover, the ease with which it can be administered during sleep, with no other action required than turning on the system at bed time, may eventually facilitate long-term compliance, which is a relevant limiting factor for the success in treating chronic disorders [35].

We have proposed that non-nociceptive somatosensory stimulation, here administered in the form of extensive and gentle mechanical vibrations, may favor the recovery of sensory balance in fibromyalgia. Our empirical results are indeed consistent with this hypothesis. However, the effect of vibrotactile stimulation on the sensory system has not been directly tested in this study. The evidence of central nervous system sensory alteration has been obtained from functional connectivity and task activation MRI [5–10]. Further neuroimaging research is thus necessary to specifically test the effects of vibrotactile stimulation on the sensory balance. Nonetheless, it is important to emphasize that symptom improvement in our study was perceived at day time with no vibrations and that their effects persisted 2 weeks after the treatment was terminated. The fact that the effects endured beyond stimulation may well indicate some functional rearrangement as opposed to a transient interference with pain signals at the entry gate [36].

Symptoms in the fibromyalgia syndrome are not independent clinical expressions but are highly interrelated. For instance, chronic pain, as a stressful situation, may favor fatigue and, conversely, fatigue may augment pain perception. Moreover, unrefreshing sleep may potentiate the feeling of both pain and fatigue. Sleep quality significantly improved in our study (Table 3). Therefore, there is a possibility that sleep improvement was to some extent a primary driver of the improved pain and fatigue. In turn, a significant degree of pain relief may well contribute to improving sleep quality. Future research may interestingly be addressed to disentangle fibromyalgia symptom interactions.

Important methodological aspects in our study included the control of therapeutic effects with strict sham and the rigorous observation of blind conditions. Our study adopted the strategy of using the same device to administer both types of intervention, presented to patients as two potentially effective options. The paraphernalia surrounding the interventions, implicating the home installation of a motorized mattress controlled by a computerized engine, was identical for both vibrotactile stimulation and sham. The placebo effect in these circumstances was indeed large and significant (e.g., first period fatigue reduction in the group sham first showed *t* = 3.6 and *p* = 0.001). However, the placebo effect was virtually nonexistent when sham was administered in the second period. This is relevant in that it may indicate that the control of treatment effects with our sham approach was not complete in this period, which is admittedly a limitation in our study. On the other hand, the data may also inform on the magnitude of placebo response and its dynamics in such an intricate chronic pain disorder as fibromyalgia, and contributes to current efforts to improve the characterization of placebo effects on pain [37, 38]. It is also noteworthy that, in studies reporting the effect of nonpharmacological treatment for chronic pain, the most common comparison has been against “usual care” with no control of the placebo effect [17].

A limitation in our study, however, precisely concerns the carryover effects. Although a complete counterbalancing in our study prevents inflation effects of either treatment option, carryover did significantly influence the magnitude of change during the second period in two ways. Firstly, symptom change was attenuated in the second period, with the previously described lack of significant placebo effects, and, secondly, the effect of vibrotactile stimulation persisted at least until week 5. Although this would interestingly suggest that the effect of vibrotactile stimulation in fibromyalgia is long-lasting, washout time should be better adjusted in future studies.

Another study limitation involves to the general problems inherent to subjective symptom measurements. It is very difficult for people, particularly for patients with chronic pain, to reliably rate the amount of perceived pain, or feelings in general [28, 29, 39]. We opted to assess outcome measures at the end of the study to facilitate the comparison of treatment effects using baseline scores as reference.

## Conclusions

The effect of gentle vibrotactile stimulation of the body on symptom relief was tested in the framework of a controlled clinical trial in fibromyalgia patients. Results showed significant reduction of pain and fatigue, and sleep quality improved despite the fact that stimulation was applied during sleep time. The degree of improvement and the easy application of our proposal would seem to be sufficiently relevant to suggest a potential role for vibrotactile stimulation in the treatment of fibromyalgia symptoms.

## Data Availability

Not applicable.

## Abbreviations

FIQ: Fibromyalgia Impact Questionnaire
HADS: Hospital Anxiety and Depression Scale
MANOVA: Multivariate analysis of variance
NRS: Numerical rating scales
